# Mechanical investigations supported by DIC of structural components intended for operation

**DOI:** 10.1038/s41598-022-22615-0

**Published:** 2022-11-09

**Authors:** T. Szymczak, A. Brodecki, Z. L. Kowalewski, D. Rudnik

**Affiliations:** 1 Department of Vehicle Type-Approval & Testing, Motor Transport Institute, 80 Jagiellonska Street, 03-301 Warsaw, Poland; 2 Department of Experimental Mechanics, Institute of Fundamental Technological Research PAN, 5B Pawinskiego Street, 02-106 Warsaw, Poland

**Keywords:** Engineering, Mechanical engineering

## Abstract

The paper deals with experimental attempts for determination of the mechanical resistance of various components subjected to static loading by analysis of displacement values in a 3D coordinate system using a non-contact testing method. The problem is studied on the basis of results from tests of the wheelchair and SUV’s wheel, supported by means of a PONTOS 5M Digital Image Correlation (DIC) system enabling determination of the patterns deflection at discretised facet measuring zones of an element. The objects tested extend a knowledge on the components’ behaviour under static loading within their loading capacity. Data collected in the experiments are expressed by variations of the resultant vector of deflection in the 3D coordinate system and images under loading and unloading stages as well. The results enabled to indicate the weakest zone in the wheelchair and to express an influence of the foot tire on the rim edge, giving necessary knowledge on the mechanical resistance of tested components. In case of the wheelchair a rear side and axle represent the weakest regions, while for the wheel a rim edge is the most loading sensitive region on the tire guard with respect to safety of the operational process. All data captured by DIC system as a function of time can be directly used for modelling and improving a suitability of the selected components since they can be employed as the limit levels during determination of safety factor. The experimental approach applied to the SUV wheel investigation can be also used as a procedure for wheels of other type vehicles tested either in laboratories of research institutions or technical universities.

## Introduction

Digital Image Correlation (DIC) technique is a method of measurement that is used in many engineering applications being in the interest of the mechanics of materials and structural components. DIC method is employed to determine a full-field strain distributions in 2D^1–4^ or 3D^5–7^ coordinate systems. It requires the specimens marked with a special spray paint creating a contrast in the form of grey-black dot pattern^5–7^. It has to be emphasised, that a type of pattern may affect the results quality significantly^8^. For example in case of the too low or too high pattern density, an erroneous strain values can be often obtained after 20 images. The grey-black dot pattern can be used not only in tests at room temperature, but also at higher temperature values, even up to 150 °C^9^. Cracks development identification is another possible application of DIC giving important knowledge related to their length, moment of opening and fracture paths direction as well^8,10,11^. The DIC method is also very suitable for compression tests of the road materials carrying out on the cylindrical specimens tested at various loading orientations. Moreover, the method enables to capture deformation of more complex specimen shapes, e.g. hexagonal shape for 3D tests^11^. It enables the efficient strain measurements at micro- and nano-scales using the high-spatial-resolution microscopes^4,12^. Thanks to the fact, that it represents the full-field displacement technique^1–3,6,13,14^, it is suitable in many practical aspects related to a time reduction of manufacturing processes, optimization of production, and the quality improvement^15–17^. Taking this into account, the following applications should be mentioned: cutting^17,18^, stamping and surfacing^17^. Among many advantages of the DIC method one can indicate an increasing speed of experimental data saving^16^. It is achieved due to elimination of the repeating redundant calculations. In typical DIC approach a speckle pattern is digitalized by means of the measurement frames having rectangular or square shapes, that are used for calculation of a displacement/strain full-field distribution in 2D or 3D coordinate systems^5,6,8,13^ without collecting other data such as displacement, velocity, and acceleration versus time at a selected point. Such approach reflects material behaviour for a chosen zones of different dimensions represented by measurement frame features. They can be distinguished by the frame size that surrounds the selected pattern of analysis for example^7,14^. At this stage, the central points of frames are used for subsequent DIC results.

The kinematic properties measurement of the complex structural components belongs to the another important group of the DIC systems applications. In comparison to the full-field DIC measurements, the method is based on the defined points of required dimensions and graphical features that are represented by black-white circles^6,7^. Details of these markers are directly introduced in the DIC software before the calibration process. The number of points for measurements is limited, and therefore, only their features should be directly taken in to account ^6,7^.

**Figure 1:**
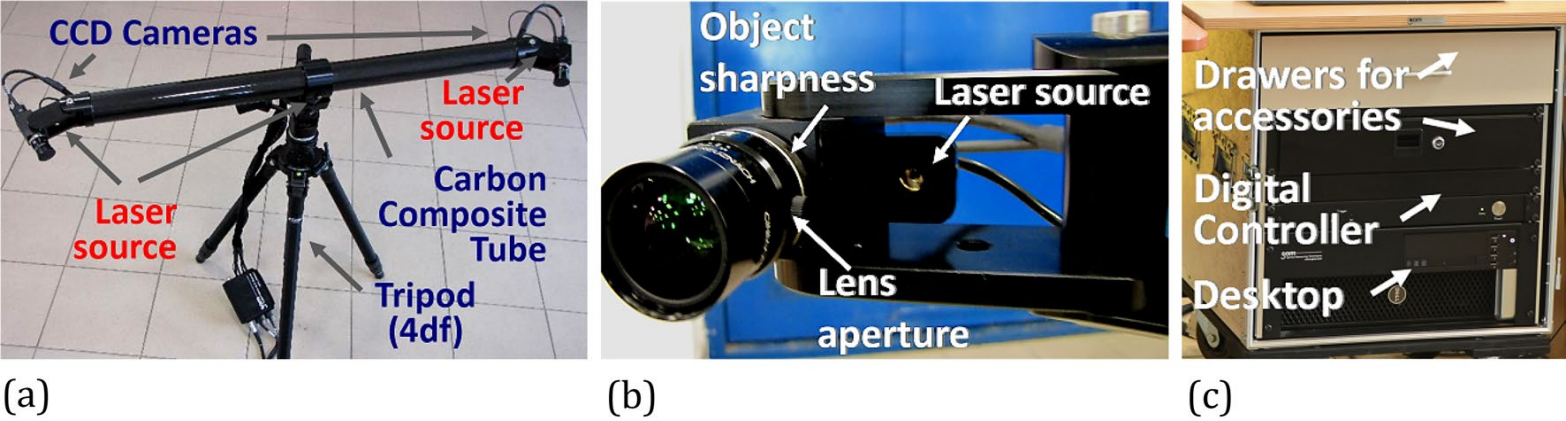
The PONTOS 5 M: (**a**) general view, (**b**) elements of the system located near the CCD camera, (**c**) control tower.

The calibration is conducted by means of the certified devices such as the plate and cross with markers coded in the form of black-white circles. It requires defined number of steps for reorientation of the objects coded with respect to cameras. In the case of plate, 13 positions are required for the calibration, while for the cross—23 ones^19^. Entire DIC system setup should contain a tube, two CCD cameras, and a laser source, Fig. 1. The cameras angle with respect of the object should be constant, equal to 25°. Usually, the objects tested are located at the same distance from both cameras^19^. It is selected on the basis of the DIC guide following calibration object (plate or cross having reference measuring points), minimum length camera support (usually equal to 500 mm), and the distance between cameras (it is constant and depends on DIC type) and measurement range (directly indicated by the system producer)^20^.

**Figure 2:**
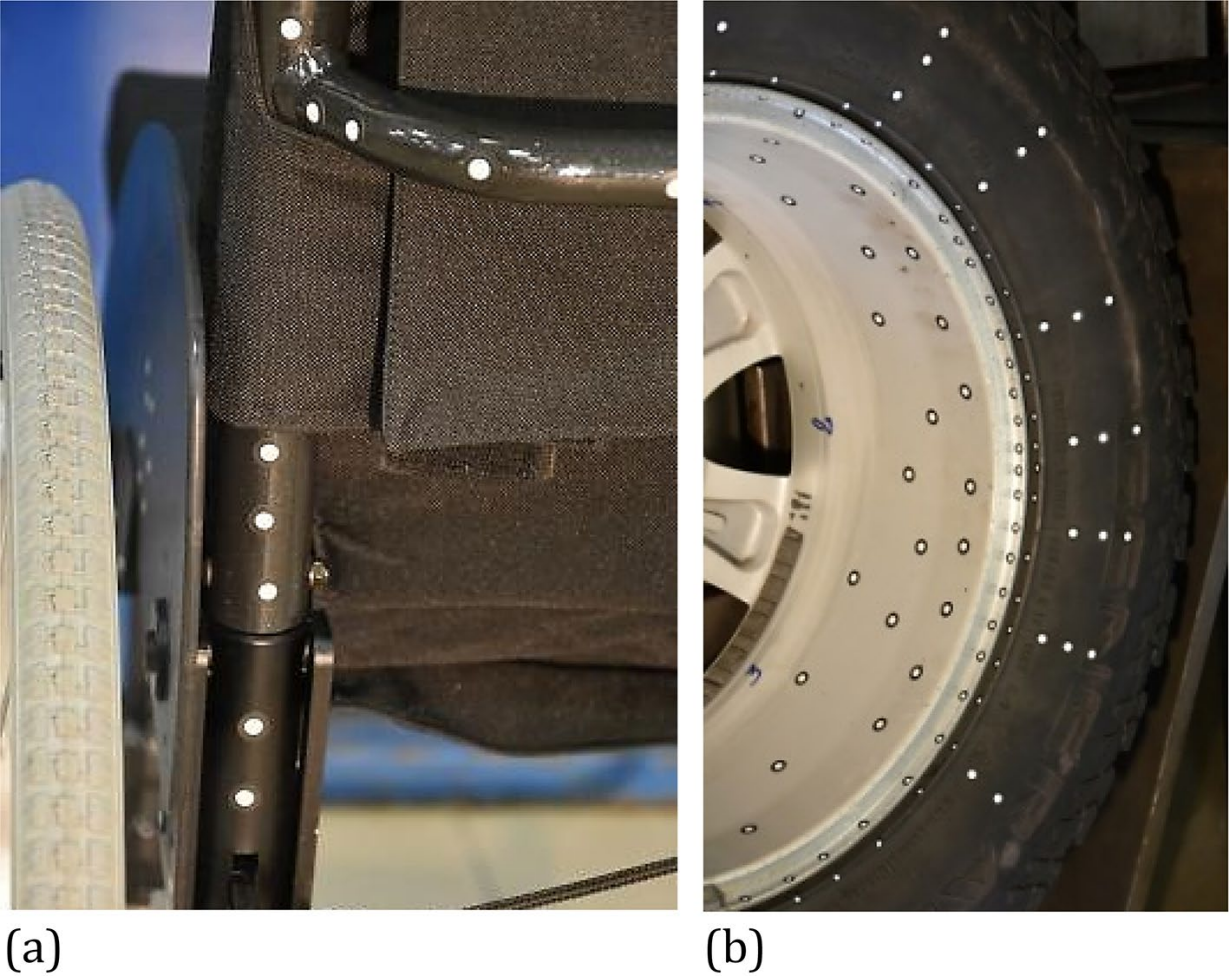
Distribution of markers on selected components of the wheelchair (**a**) and entire wheel (**b**) tested using a PONTOS 5 M DIC system.

The laser source is used to calibrate the device by means of three laser beams focused on the selected spot in the central point of 3D measurement space. The markers attributed to the selected zones are tracked by the DIC system Fig. 2. They are very helpful in vehicle doors quality assessments^21^. Moreover, they can be successfully used for a huge engineering structures such as wind turbines for example^22^. In such cases, the markers enable determination of a deviation in 0 × direction for the three blades independently, and as a consequence, differences between the collected results. Also, the vehicle wheels of a car can be easily covered by the markers under dynamic tests, thus enabling analysis of the following axis and tire deflection and wheel amplitude at different values of speed^23^. DIC applications in biomedicine offer a new opportunities in diagnostics, where the feet deflection determination in the 3D coordinate system can serve as a classical example^24^. The results of biomedical tests presented in^25^ show another example of DIC usage, expressed by the foot arch measurements. Interesting DIC application is also presented in^26,27^ where markers were recommended for determination of the kinematic features of a phantom head.

The markers take the form of the white-black circular spots of various dimensions, ranging from 0.4 to 25 mm^7^. They were directly glued to the surface of the research object. In terms of lighting conditions, the top layers of selected markers have a fluorescent cover and they are distributed with respect to the major axis of the component to be tested, Fig. 2a. Their distribution is strongly related to the major axes of the components. For objects without a significant major axis, the markers can be distributed in the stochastic manner, Fig. 2b (rim), or in the expected deformation zones of the components edges tested, Fig. 2b (tire). Depending on the object tested the following diameters are usually applied: 5 mm in case of the wheelchair, while for the SUV wheel 3 mm in case of the rim edge, 5 mm for the rim inner zone and tire), Fig. 2.

**Figure 3:**
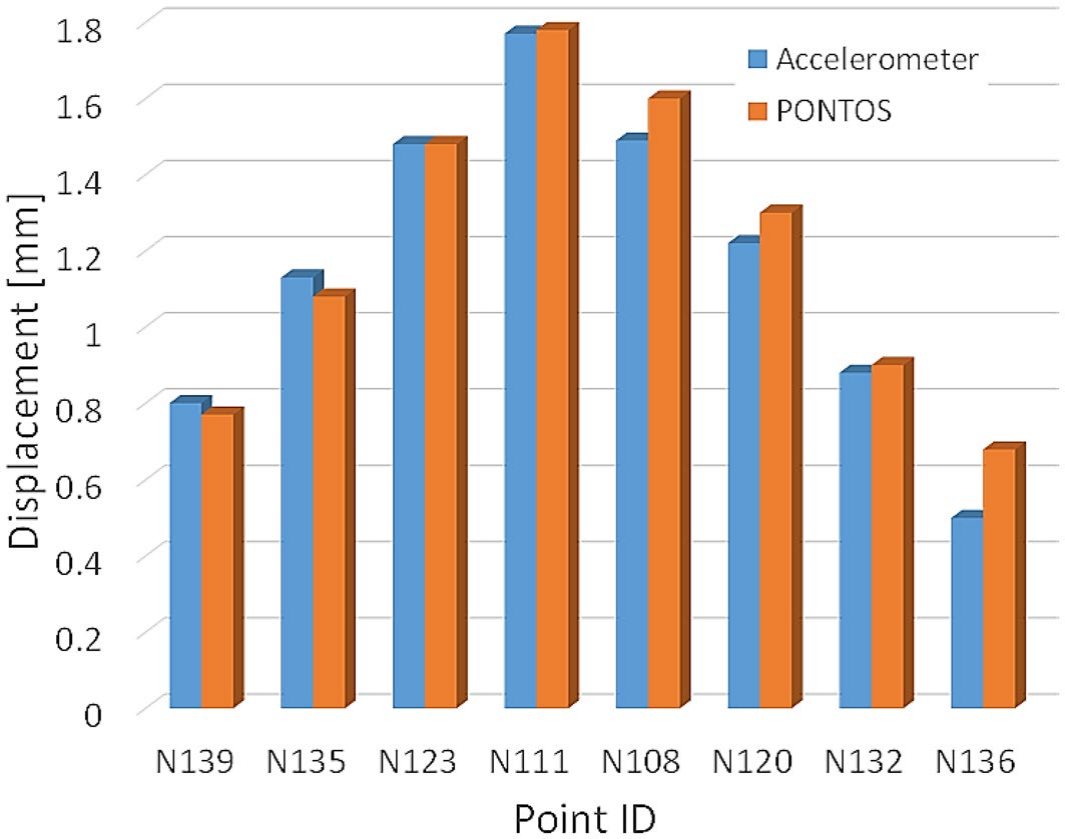
Displacement determined by means of accelerometer and PONTOS DIC system^30^.

The DIC system can be used for both the static and dynamic testing condition^21^. An analysis of the available literature on the DIC systems usage for the assessment of structural components indicates that this method is suitable for tests carried out under cyclic loading^28–31^. This is confirmed by data shown in Fig. 3 where displacement values collected by means of two devices, i.e. accelerometer and PONTOS, are presented. An analysis of the results leads to the remark that the DIC system can be even suitable for displacement measurements of objects smaller than 1 mm. One can notice, that both methods are complementary, since the results obtained by means of them are almost the same, and therefore, they can be used interchangeably, dependently on the resources of the laboratory.

**Figure 4:**
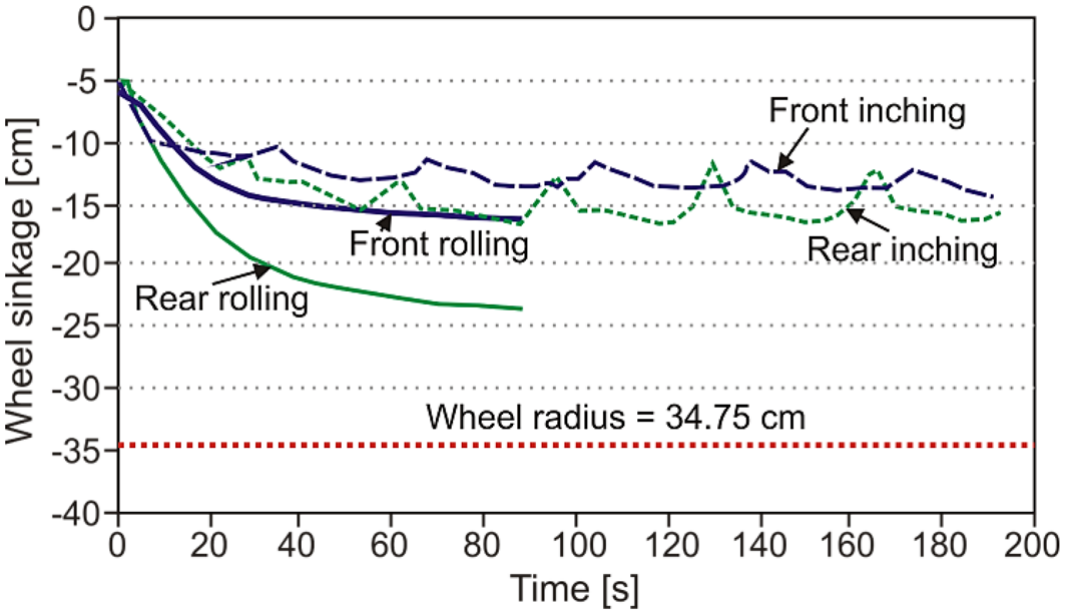
Wheel sinkage of the Scarab NASA rover in DIC measurements^32^.

The DIC system can be employed for determination of a shape of the component^30^, movement (Fig. 4)^32^, and linear velocity^28^ under rotation. As it can be noticed in Fig. 4, an advantage of the DIC system is clearly visible. This is the measurement range enabling observation of the whole vehicle, taking front and rear wheels^32^. Additionally, the tested object can be analysed at the different types of movement like rolling or inching, based on the wheel sinkage courses. The fluctuations taking place during the inching cycle are clearly visible in Fig. 4, thus confirming a good sensitivity of the DIC method in such investigations.

DIC system was also effectively used for the in-situ pavement testing to evaluate its quality with respect to the conservation process^33^. In this case, the head of the measuring device was aimed directly at the examined surface with the pattern applied using a multi-jointed tripod.

Despite just presented examples, a number of publications devoted to DIC application at measurement points is still insufficient. Therefore, the aim of this paper is to provide new attempts for the DIC system usage in the deformation analysis of structural components that have a specific geometry and are subjected to static loading conditions which take place during the wheelchair and SUV wheel exploitation. This is directly related to the practical aspects, i.e. safety increase for disabled persons when using the wheelchairs and drivers of SUV vehicles. Although both tested objects are commonly exploited, they were not tested using the DIC technique so far. Therefore, this can be indicated as a new approach for testing of such components. In addition, investigation of the SUV wheel was carried out as the order of the military branch for establishing causes of the circumferential fractures of the rim edge.

## Details of the experimental procedure

**Figure 5:**
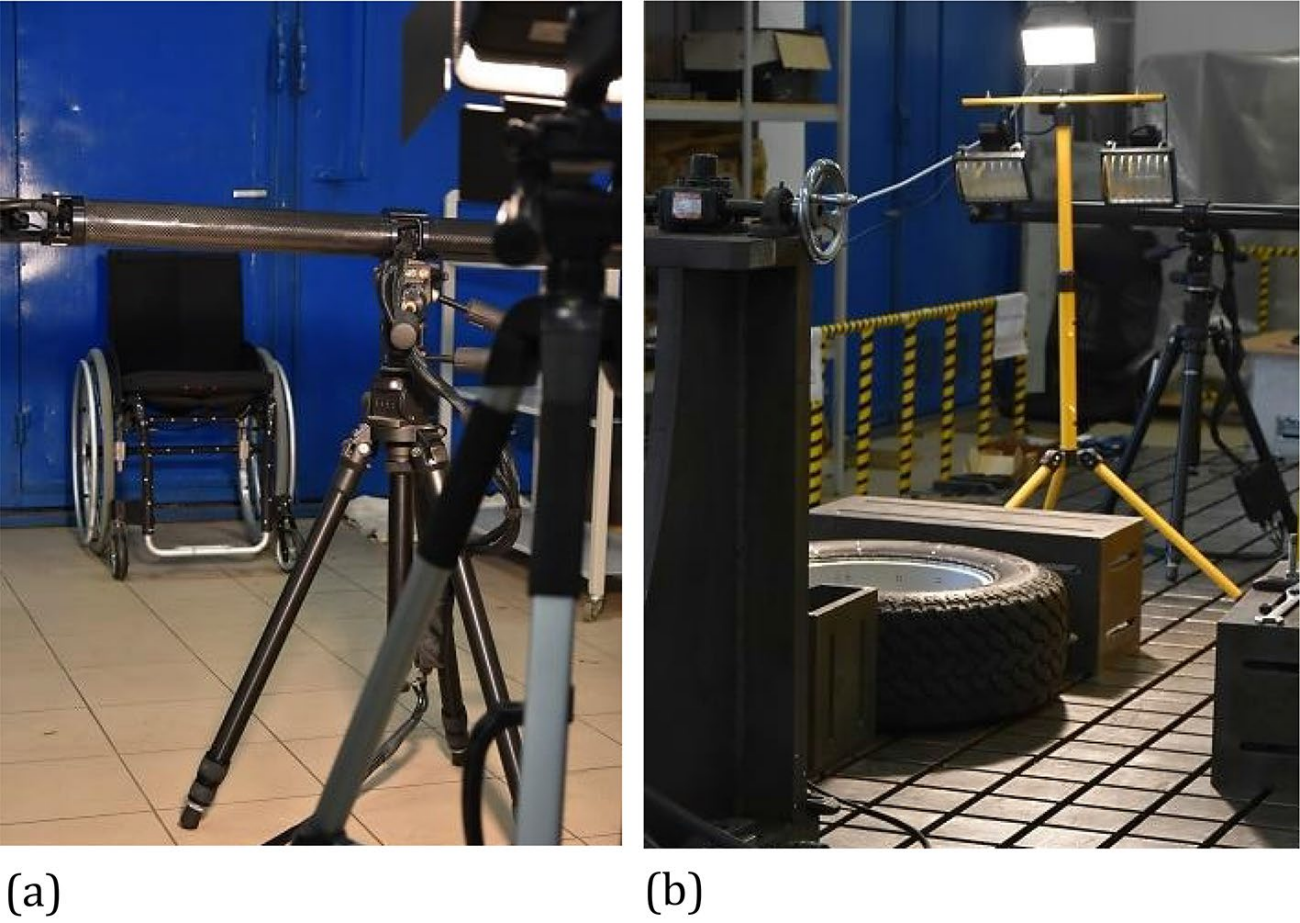
PONTOS 5 M DIC system used in experiments: (**a**) wheelchair; (**b**) SUV wheel.

Measurements carried out by means of DIC system are strongly dependent on the lightening conditions, because they have a direct influence on the number of measurement points taken for analysis. If the lightening conditions are insufficient for DIC measurements, an additional light source should be used, Fig. 5. It can be checked in the DIC software during preliminary calculations of displacement coordinates.

Positioning of the extra light is dependent on the testing station design, dimensions and location of the object in question. If a height of the tested object is close to that of the DIC’s measuring tube, then it is recommended to locate the light source behind the station, Fig. 5a. In the opposite case, the lamp should be located between the tested object and DIC system, Fig. 5b. It is recommended to use the light source with its power adjustment.

Two different objects were tested: a wheelchair (Fig. 5a) and a wheel from the Sport Utility Vehicle (SUV) (Fig. 5b). The wheelchair was made of steel and had a typical construction. A rim of the wheel was manufactured from aluminium alloy (42,100 (AlSi7Mg0.3)) by means of a hybrid procedure including casting and quasi-forging processes.

**Figure 6:**
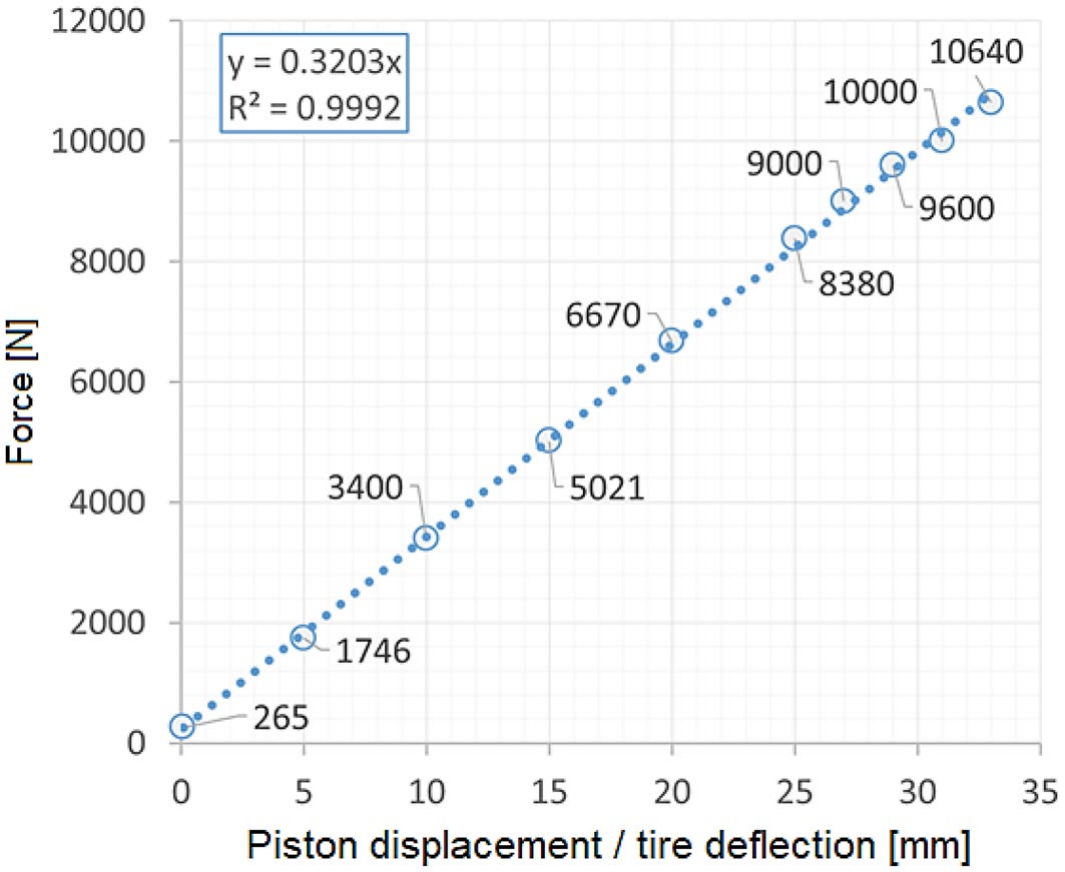
Force versus the tire deflection of the SUV wheel.

**Figure 7:**
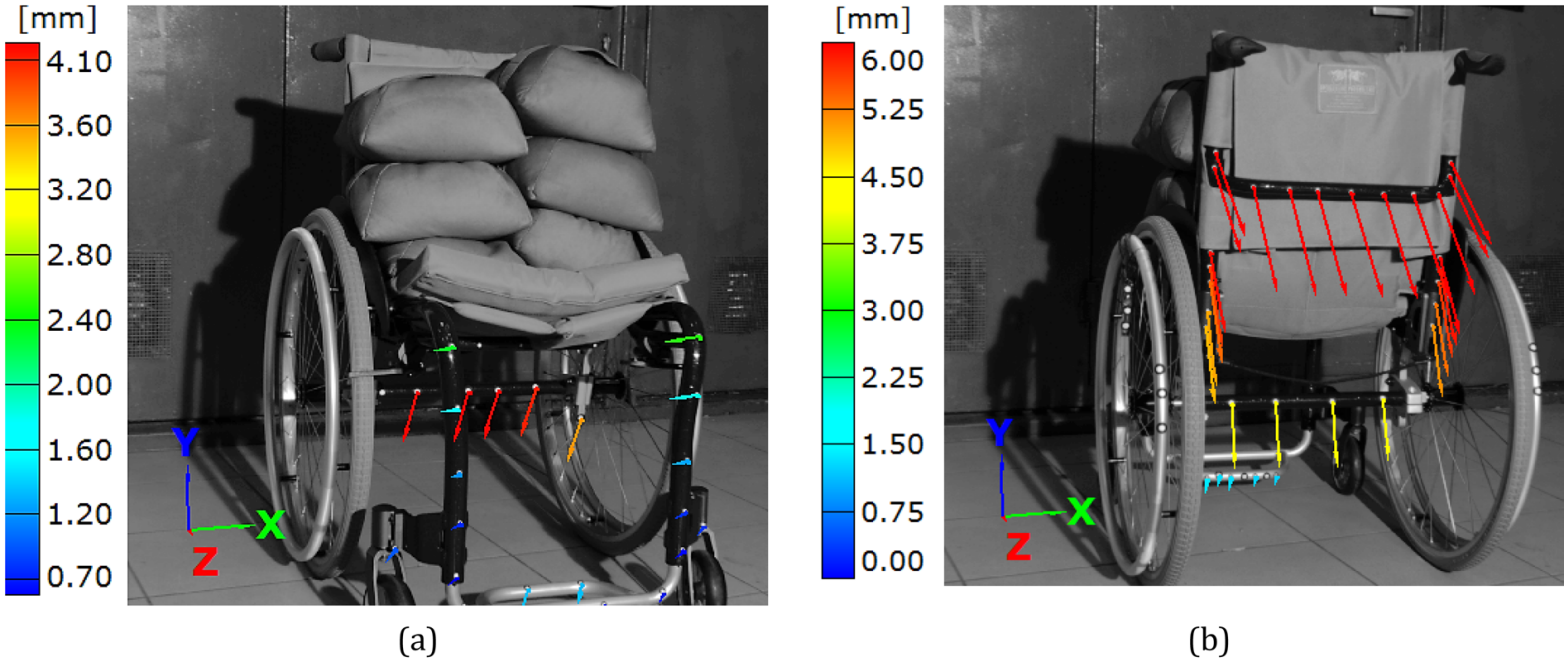
Distribution of the resultant vectors of deflection in the wheelchair loaded by the force of 1.5 kN in views from the front (**a**), and rear (**b**) sides, respectively.

**Figure 8:**
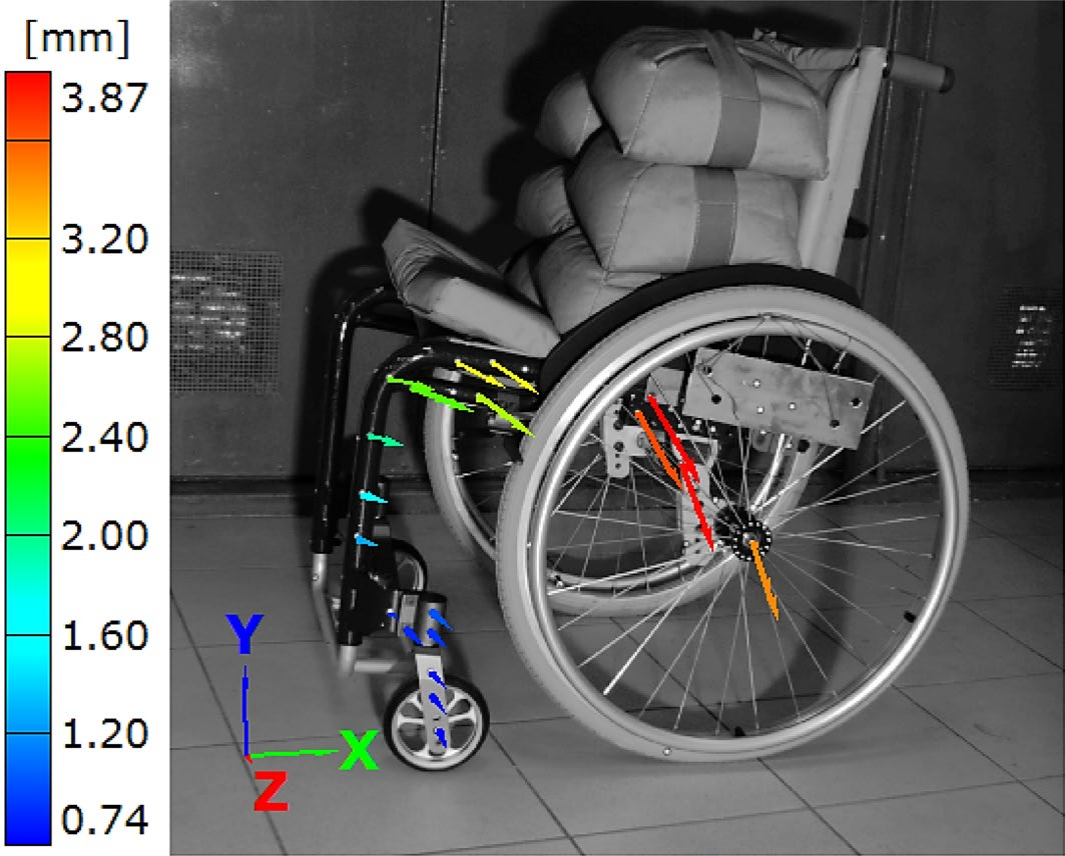
Distribution of the resultant vectors of deflection in the wheelchair loaded by the force of 1.5 kN from side view.

All tests were conducted at room temperature under static loading. They were supported by a PONTOS 5 M (Digital Image Correlation system) for the measurement of displacement components. Due to the limited lighting in the test region, additional lamps were applied, and as a consequence, the lighting system enabled better observation of the measuring points by DIC, Fig. 5. For the wheelchair, the loading was realised by means of 25 kg bags filled with a metal shot, Figs. 7 and 8. A Saginomiya servo-hydraulic actuator with the loading capacity of ± 30 kN and digital controller IST (Instron Structural Testing) was chosen for examining the SUV wheel, Fig. 6.

In case of the wheelchair, the force of 1.5 kN (Fig. 7b) was selected in order to reflect the maximum weight of the wheelchair user. In case of the SUV wheel, it was equal to 11 kN (Fig. 6). The loading path contained 10 steps. Their number increased towards the end of test. At each loading point, a displacement value of the actuator’s piston was registered to follow the tire deflection.

Assessment of the tested object was carried out on the basis of displacement variations in the 3D coordinate system under both loading and unloading stages. DIC data were collected manually after levelling value of loading by means a drain hose.

## Results and discussion

The results of DIC experiments are presented in the form of images with deflection vectors, fringe plots and diagrams of deflection/displacement variations versus time, Figs. 7, 8, 9, 10, 11, 12, 13, 14, 15, 16 and 17.

**Figure 9:**
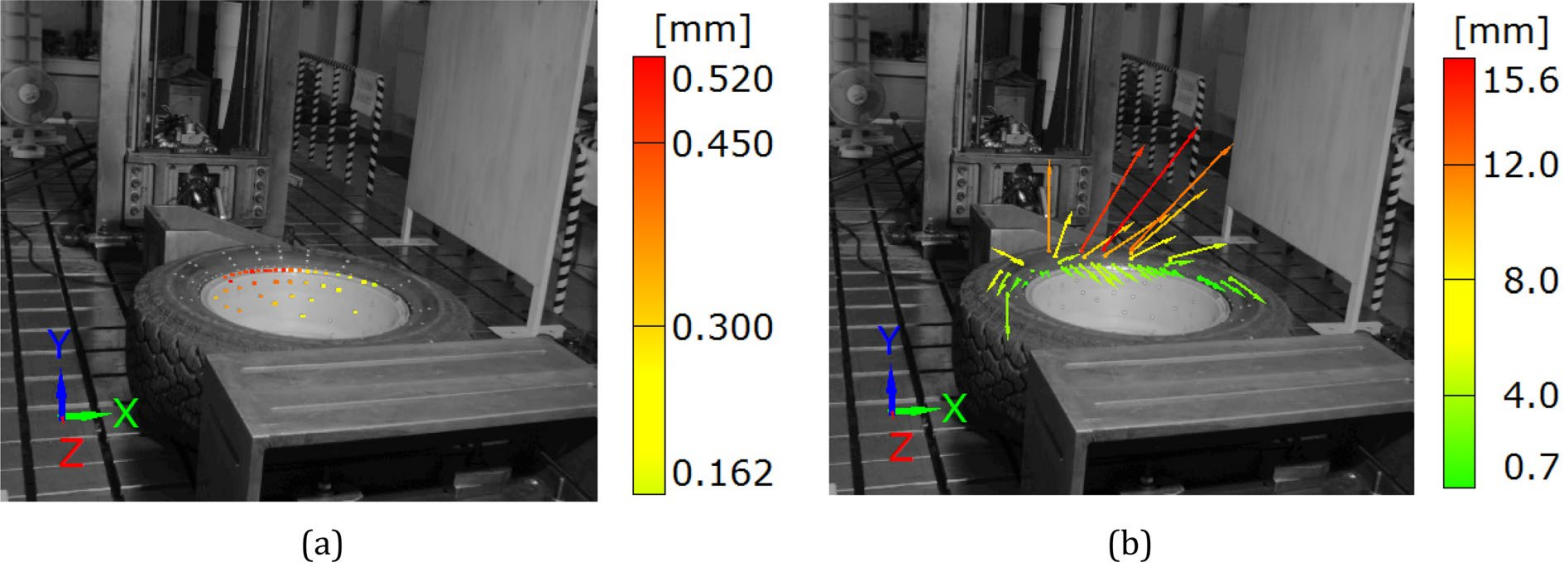
Distributions of the resultant vectors of deflection in the SUV wheel under the force of 11 kN for the rim (**a**) and tire (**b**).

**Figure 10:**
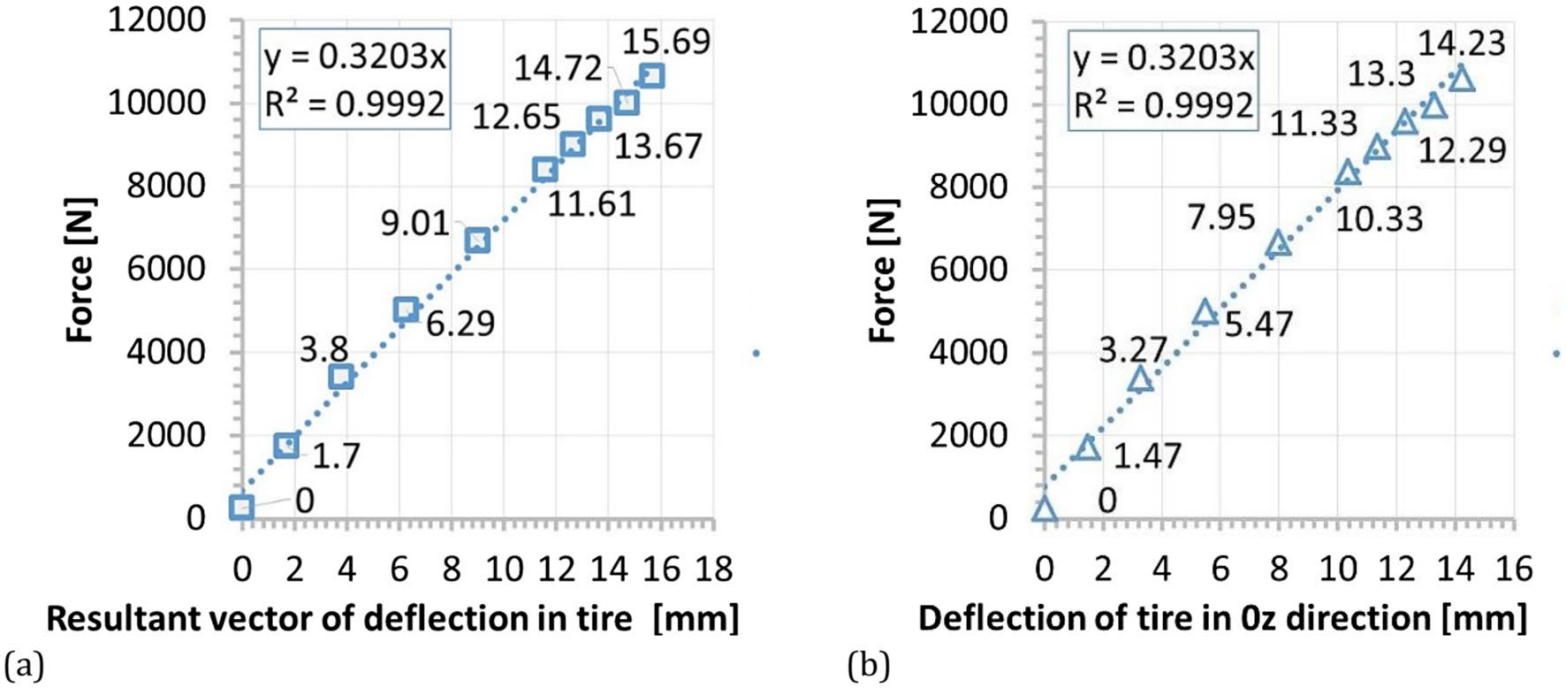
Force versus: (**a**) resultant vector of deflection in tire; (**b**) deflection of tire in the 0z direction.

**Figure 11:**
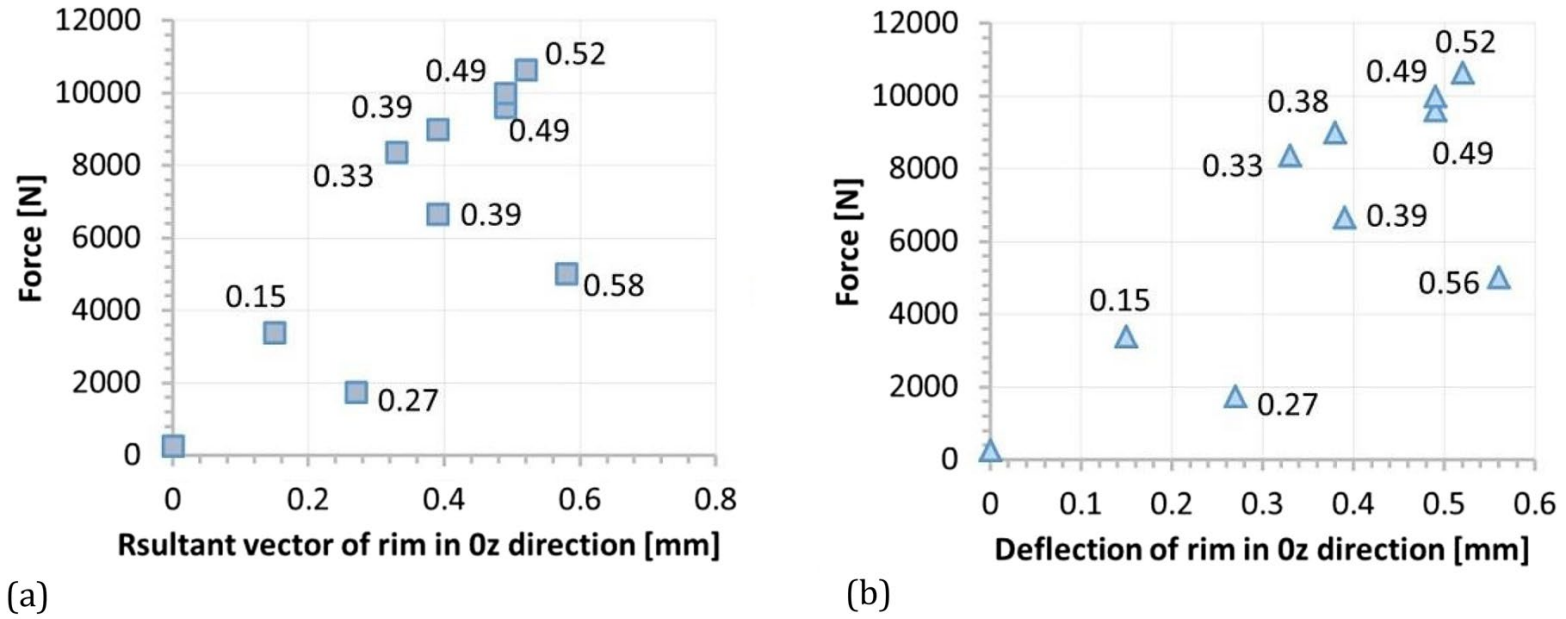
Force versus: (**a**) resultant vector of deflection, (**b**) deflection of tire in the 0z direction for rim.

**Figure 12:**
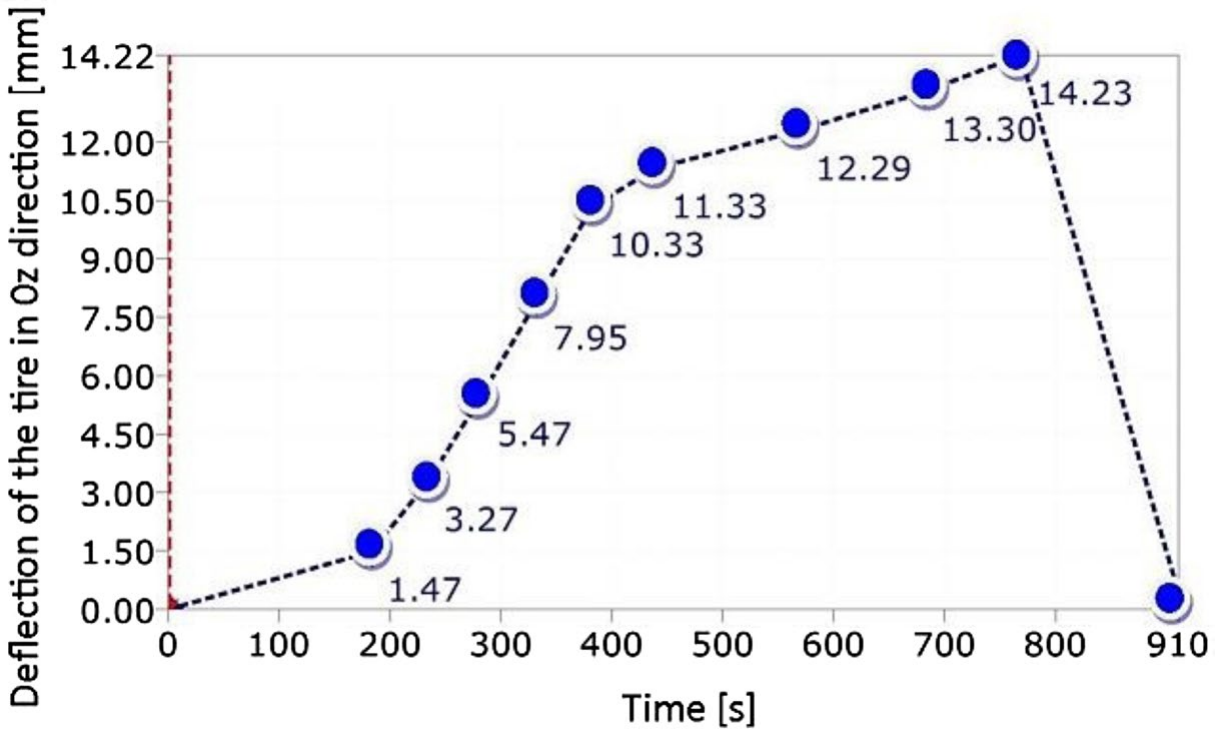
Resultant vector of deflection for the SUV tire under static loading of up to 11 kN.

**Figure 13:**
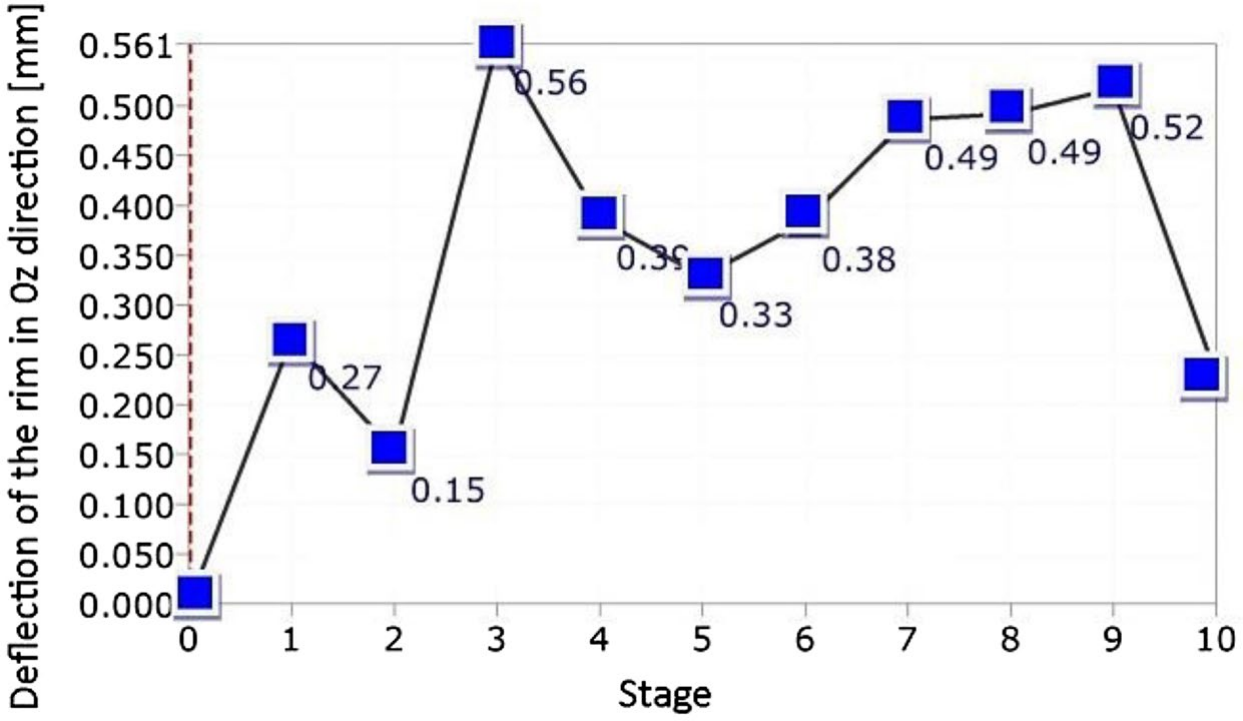
Resultant vector of deflection for the SUV wheel’s rim under static loading of up to 11 kN.

**Figure 14:**
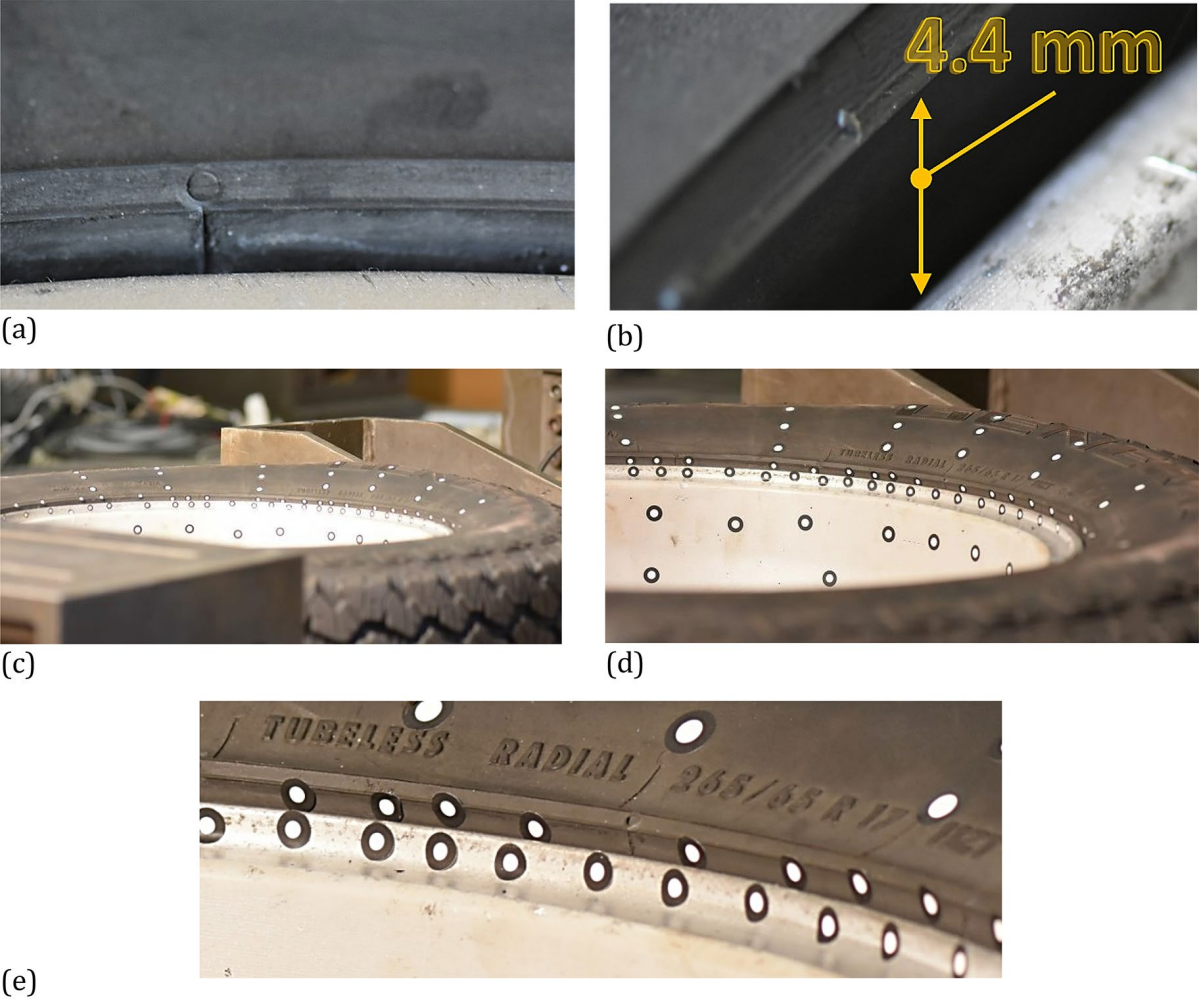
The wheel: (**a**) and (**b**) the tire guard and rim edge before loading; (**c**), (d), (e) under force of 11 kN: general view, magnified view of deflected tire and edge of the rim, respectively.

**Figure 15:**
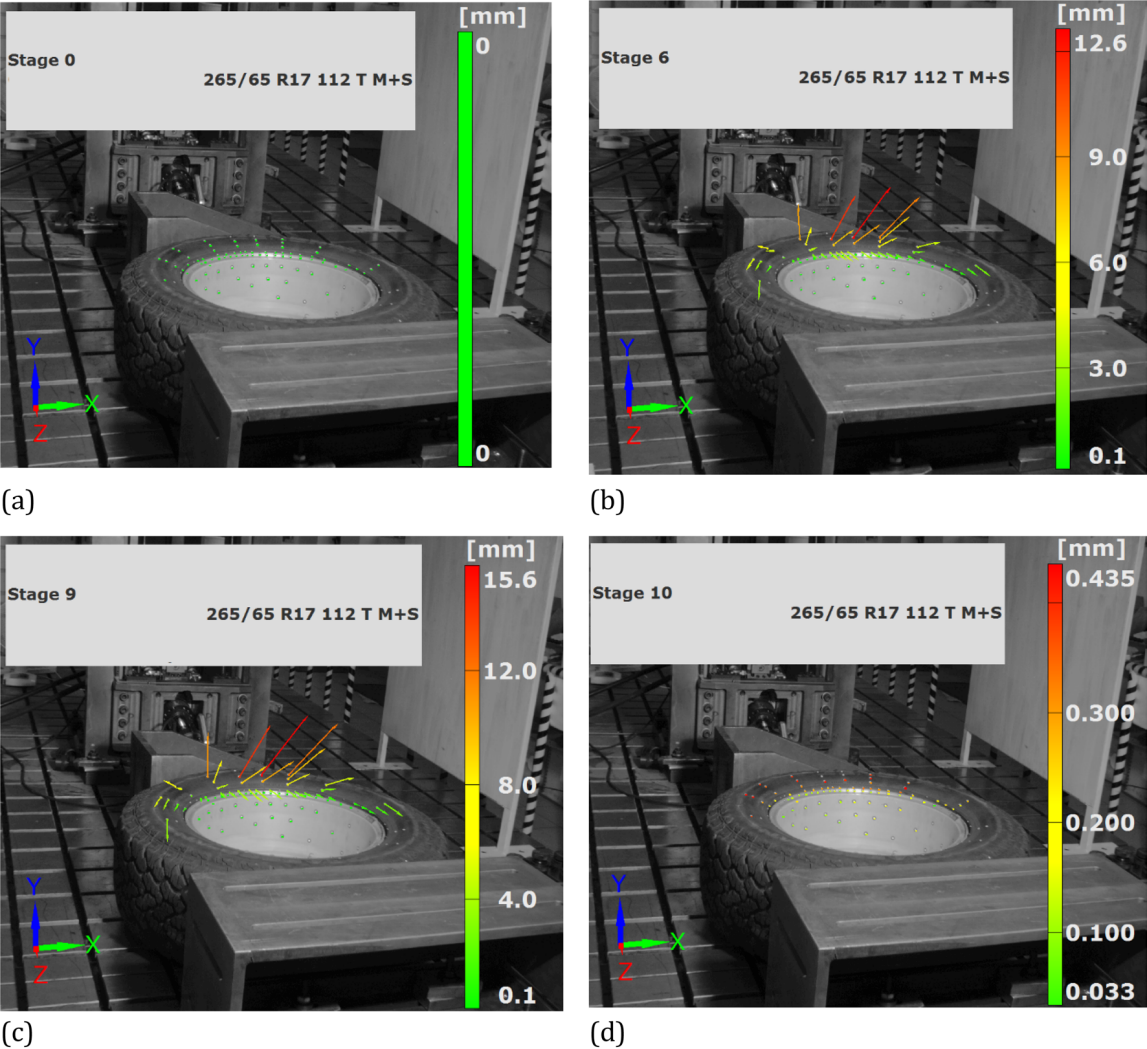
Resultant vector at different stages of loading path: (**a**) 0 kN, (**b**) 9 kN, (**c**) 11 kN, (**d**) unloading.

**Figure 16:**
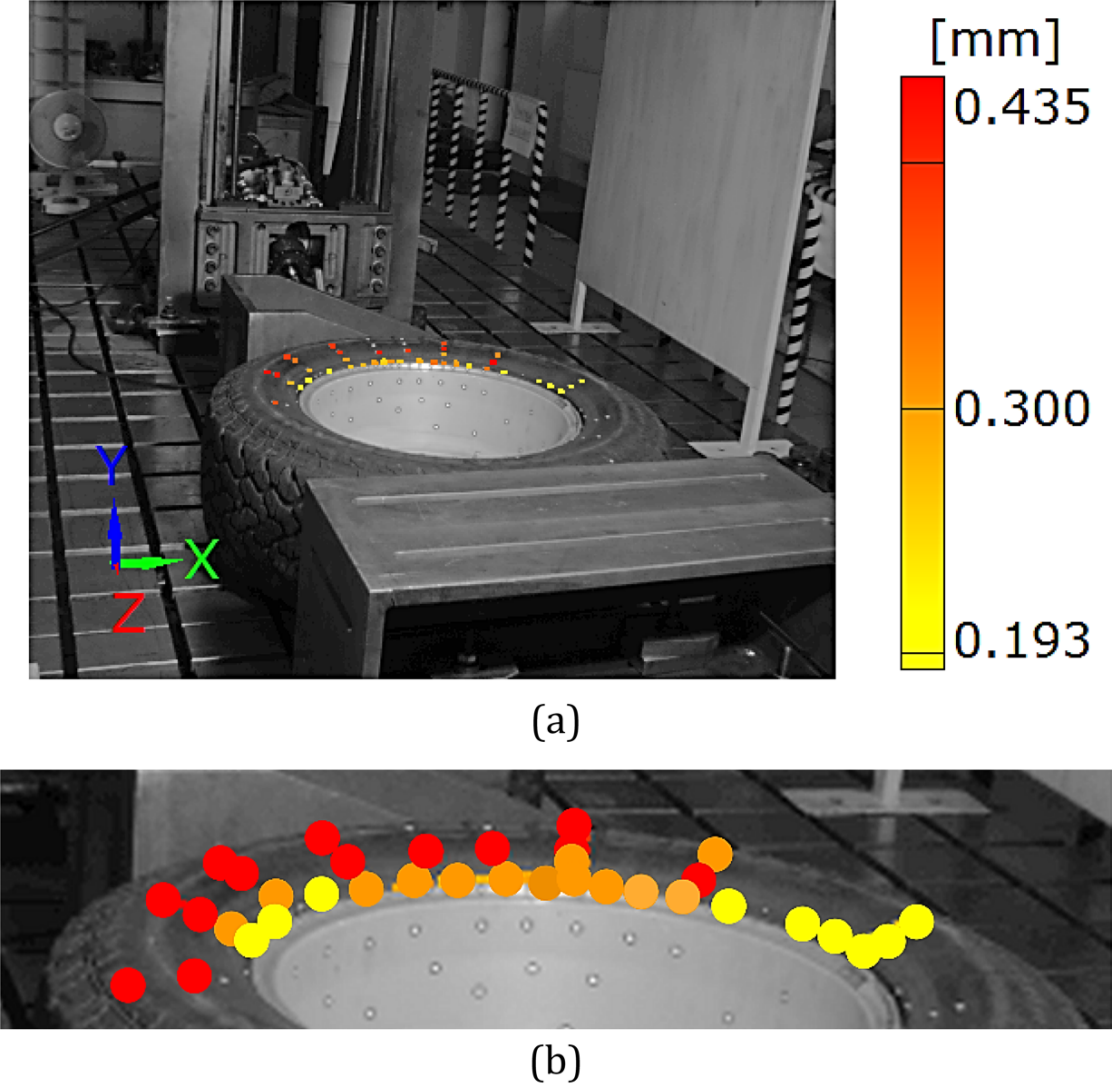
Distribution of the resultant vectors of deflection for the SUV tire after unloading: (**a**) general view, (**b**) magnified view of the tire.

### The wheel chair under static force equal to 1.5 kN

In case of the wheelchair, DIC observations were presented for various orientations with respect to the tested object, Figs. 7 and 8. Different arrangements of the object enabled capturing deflections in the 3D coordinate system. Analysis of them allowed identification of the possible collapse of the tested object, Figs. 7a and 8. Moreover, the largest deflection and its variations were easily indicated.

As it can be noticed, the maximum value of the deflection (6 mm) was recorded for the rear headband, Fig. 7b. In comparison to the results for the wheel axel, Fig. 8, this value was 2.5 times greater. It indicates significant differences between the stiffness of the components for both objects in question. Based on the wheelchair data the following conclusion can be formulated, namely, the object should be redesigned using alternatively, elements of varying cross-sections or other materials.

It has to be emphasised, that this kind of experimental results cannot be find in the available literature, therefore, they can be treated as a new result, suitable for wheelchair design, operation and selection for people with disabilities. Although the results for the wheelchair were successfully determined for all directions of the 3D coordinate system, some difficulties were also noticed. They were related to the re-orientation of the tested object with respect to its position in the DIC system, which should be located taking the central point during the PONTOS 5 M calibration. In the opposite case, data for the selected region of a wheelchair could not be recorded.

### The SUV wheel under monotonically increasing force up to 11 kN

**Figure 17:**
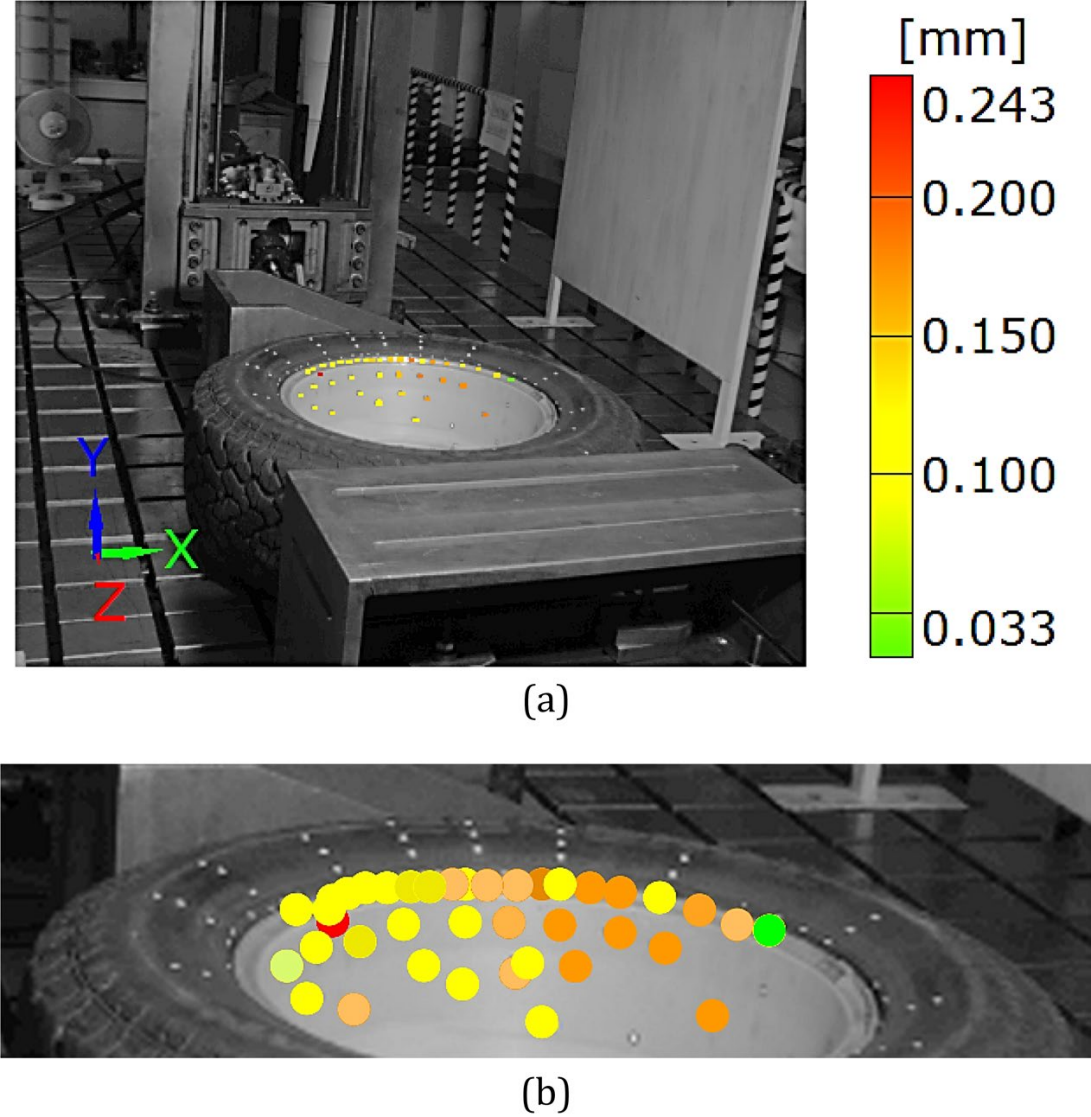
Distribution of the resultant vectors of deflection for the SUV rim after unloading: (**a**) general view, (**b**) magnified view of the tire.

Testing of the SUV wheel showed the suitability of the DIC system in experiments where the components are deflected with respect to all axes of the 3D system, even for large displacements, Figs. 9, 10, 11, 12, 13, 14, 15, 16 and 17. DIC technique applicability was also proved for very small deflection values, Figs. 9a and Figs. 16, 17. Both these aspects are discussed in the further part of this section.

An analysis of experimental data identified a linear relationship between the acting force and displacement for the whole range of values determined in the case of tire investigations, Fig. 10. As it can be easily illustrated a deflection component playing a main role in the tire deformation can be identified, Fig. 10b.

It was found, that no typical tendency was observed between force and deflection in the case rim. Here, a strong stochastic distribution of the results was obtained. Moreover, the deflection values were very small, not exceeding 0.6 mm, Fig. 11. It means, that the loading of the wheel was generally carried out by the tire, and therefore, the rim was only subjected to insignificant deflection, Figs. 12, 13, 14c–e, 15. Although the loading stage is very important for a precise assessment of the mechanical resistance of the object tested, also the unloading stage plays an essential role in taking into account the permanent deformation occurring in the rim, Figs. 13, and 17. As it can be noticed in Figs. 13, 15, even minimal deflection differences can be indicated.

Taking into account the results obtained for the wheel, an influence of the tire on the rim can be identified. It is expressed by the permanent deflection of the rim reaching a value of 0.243 mm, Figs. 13 and 17. It was caused by the tire guard, because it was not matched to the edge rim tested. Based on the results for the wheel as a whole, the influence of the difference between the height of the tire guard and the rim edge was noticed, Fig. 15. For that case the vector value at the edge of rim was within the range of 3 ÷ 4 mm, thus expressing the influence of tire on the rim. A permanent deflection of the tire was also noticed, Fig. 16.

Among many advantages of the DIC system, one can indicate a possibility of data presentation as a function of time either under loading (Figs. 12, 13, 15) or unloading (Figs. 15d, 16, 17). Data from loading and unloading enables to determine the permanent deflection.

The unloading stage revealed a permanent deformation of the tire (Figs. 12, 16) and rim (Figs. 13, 17) expressed by the following values 0.4 and 0.2 mm, respectively. In the case of tire, the value of permanent deformation determined is not significant, because the component dimensions are significantly higher than the result captured. Deformations of the rim and its edge of 0.2 and 3.67 mm, respectively, are more important due to amount of their ratio equal to 5.5% what indicates, that the rim edge did not fulfill the nominal technical feature. It means, that the region was subjected to fatigue of the rim’s material. Taking into account all data collected, the main cause of the rim deformation can be identified. It was directly connected with the poor choice of the rim and tire, that led to appearance of an additional loading component in the form of the bending moment (Fig. 9, 15). This is due to incompatibility between the tire foot and rim edge, Fig. 14. It indicates that the tire and rim selection was not carried out properly, even though the two components match well to the basic technical parameters. One can conclude, that the pairing of the tire and rim should not be done freely, but always must be done according to the recommendations of the tire manufacturer.

Taking all stages of the SUV wheel examination some difficulties can be also found. They were related to the lighting system and sloping of the measuring system with respect to the tested object. A weak lighting makes it impossible to recognize all markers, thus limiting the number of results for component tested under loading. In the opposite case, reflections from the rim can appear, that led to the same effect. Therefore, several combinations of the light incident on the object tested have to be considered in order to record a significant number of measurement points. A sloping measurement tube of the PONTOS 5M had the same role as the lightning device and it required many experimental steps to obtain an acceptable number of measuring points.

The SUV wheel testing enabled to propose the procedure for examining an influence of the tire guard on the rim edge. It can be directly used in either expert opinions or scientific attempts in order to avoid an unexpected accidents expressed by rim deformation and brittle-ductile circumferential fracture. The procedure contains the following steps:

1. mounting the wheel with the use of strength screws to the T-slots test platform,
2. sticking the markers on the tire and rim. In the case of a tire, the measurement points should be radially arranged while in the case of a rim both variants should be used i.e. and circumferentially for a rim edge and radially for a middle part of the rim. Two different sizes of markers can be used i.e. a smaller and bigger for a rim and tire, respectively,
3. calibration of DIC system by light source for collection of the sufficient number of markers,
4. application of a flat stamp between the actuator rod and wheel of dimensions sufficient to cover the width of tire and large area of the circumference, thus minimizing the occurrence of a concentrated load,
5. usage of force signal as the control parameter for the servo-hydraulic actuator enables to establish a load values up to the tire ultimate load. It is recommended to select not less than 10 loading levels,
6. each loading stage should be captured by DIC system and macro-photography technique to keep a distance in terms of the safety of the research team,
7. an unloading stage should be performed slowly without a dynamic response of a wheel. It is recommended to capture DIC photos after unloading, which can be determined by 30 min.

## Summary

The paper presents the results from static loading tests of the wheelchair and SUV’s wheel, that were supported by means of the PONTOS 5 M Digital Image Correlation (DIC) system.

DIC method enabled to reflect the important differences in responses of elements and their immediate surroundings under loading and unloading states. The rear bar of the back was identified as the weakest part of the wheelchair. It exhibited the highest value of deflection.

In case of the wheel, the edge rim represented the weakest zone. It was assessed by differences between measurements of DIC characterizing this region and the tire guard. Such result indicates that a selection of the tire and wheel should be conducted on the basis of the manufacturer’s recommendation, and definitely, it cannot be done based solely on the knowledge related to the general dimensions and technical features.

DIC measurements of the multi-element components were conducted at various orientations of the 3D coordinate system. This is because some regions of the tested object may not be well visible due to the shadow zones from other parts or obscuration by other elements of the object in question.

The research emphasises an importance of the additional lighting source. It enables identification of more measuring points if their diameter is very small compared to the distance between DIC system and the object tested. This should be directly checked in the reference image by performing calculations of displacement values before loading. From the scientific and practical points of view, the lightening should ensure observation of all measurement points, because some of them can be lost during loading due to significant deformation of subcomponents, that leads to a shape change of the selected reference measurement points from circular to elliptical, or due to possible light reflections appearing as an effect of important local geometry changes of the object tested.

This work reflects an additional experimental approach related to the DIC technique usage. It can be formulated as a quantification of the relationship between the intensity level of a light source and the number of measurement points and the quality of the data.

## Data Availability

The datasets generated and/or analysed during the current study are not publicly available due [PROJECT RESULTS FOR A MANUFACTURER AND EXPERTISE DATA FOR MILITARY INDUSTRY] but are available from the corresponding author on reasonable request.
